# Rett syndrome complicated by diabetes mellitus type 1

**DOI:** 10.1530/EDM-24-0010

**Published:** 2025-02-05

**Authors:** Yasutaka Kuniyoshi, Satoru Takahashi

**Affiliations:** ^1^ Department of Paediatrics, Tsugaruhoken Medical COOP Kensei Hospital, Hirosaki, Japan; ^2^ Department of Social Services and Healthcare Management, International University of Health and Welfare, Otawara, Japan; ^3^ Department of Paediatrics, Asahikawa Medical University, Asahikawa, Japan

**Keywords:** diabetes, paediatric endocrinology

## Abstract

**Summary:**

Rett syndrome (RS) is an X-linked neurodevelopmental disorder primarily affecting females. RS and diabetes mellitus (DM) type 1 are rare disorders with distinct etiologies. Although some cases of RS complicated by DM type 1 have been reported, the association between these distinct conditions is poorly understood and warrants further studies to elucidate the underlying mechanisms and inform clinical management. We report the case of a 10-year-old girl diagnosed with RS and DM type 1. The patient initially presented at 3 years of age with polydipsia, polyuria and decreased appetite over several weeks. Physical examination showed signs of dehydration, and laboratory evaluation revealed hyperglycemia, elevated HbA1c, glycosuria, ketonuria and low C-peptide levels. Anti-glutamic acid decarboxylase antibodies were positive, confirming autoimmune DM type 1. Fluid resuscitation and insulin therapy were initiated with good glycemic control on continuous subcutaneous insulin infusion. A review of her history revealed normal early developmental milestones, including the onset of stereotypical hand movements at 3 years, communication impairment and seizures at 4 years and a diagnosis of autism spectrum disorder. At 10 years of age, genetic testing revealed a pathogenic MECP2 mutation. Clinical features, including breathing abnormalities, bruxism, abnormal tone and inappropriate laughing, met the diagnostic criteria for RS. This is the reported first case of RS with a confirmed MECP2 mutation complicated by DM type 1. Our case report contributes to the increasing evidence supporting the potential association between RS and DM type 1.

**Learning points:**

- There is a possible link between RS and DM type 1.
- This is the first case report of RS with a confirmed MECP2 mutation complicated by DM type 1.
- In cases where patients with RS develop diabetic ketoacidosis, it may manifest as mild acidosis or normal pH despite the presence of high blood sugar levels and dehydration.

## Background

Rett syndrome (RS) is a rare X-linked neurodevelopmental disorder that predominantly affects females, leading to impaired cognitive, motor and social functions (1). On the contrary, diabetes mellitus (DM) type 1 is an autoimmune disease characterized by the destruction of pancreatic beta-cells, ultimately resulting in insulin deficiency. Although RS and DM type 1 have distinct etiologies, there are a few case reports documenting their co-occurrence. The underlying mechanism of this association remains unelucidated, and clarification may have critical clinical implications for the diagnosis and management of both conditions. Here, we present a case of RS complicated by DM type 1. In addition, we compared the patient characteristics and clinical findings of our case with the other cases reported in the literature.

## Case presentation

A 3-year-old female presented to our department with symptoms of polydipsia, polyuria and loss of appetite over the past several weeks. She had no significant past medical history and was not taking any medications. There was no family history of DM. Physical examination revealed that she was dehydrated, with a heart rate of 125 beats per minute, respiratory rate of 40 breaths per minute, percutaneous oxygen saturation of 99% and a body temperature of 37.2 °C.

## Investigation

Laboratory tests revealed hyperglycemia (blood glucose: 1,091 mg/dL) and elevated HbA1c (10.6%). Venous pH fell within normal limits (7.354), while the bicarbonate level was low (14.1 mmol/L). Urinalysis showed glycosuria, ketonuria and a low level of C-peptide (2.1 μg/mL). Further investigation revealed that the patient was positive for anti-glutamic acid decarboxylase antibody.

## Treatment

She was promptly started on fluid resuscitation and insulin therapy. Continuous subcutaneous insulin infusion was administered after the acute phase, and blood glucose levels were well controlled.

## Outcome and follow-up

**Figure 1:**
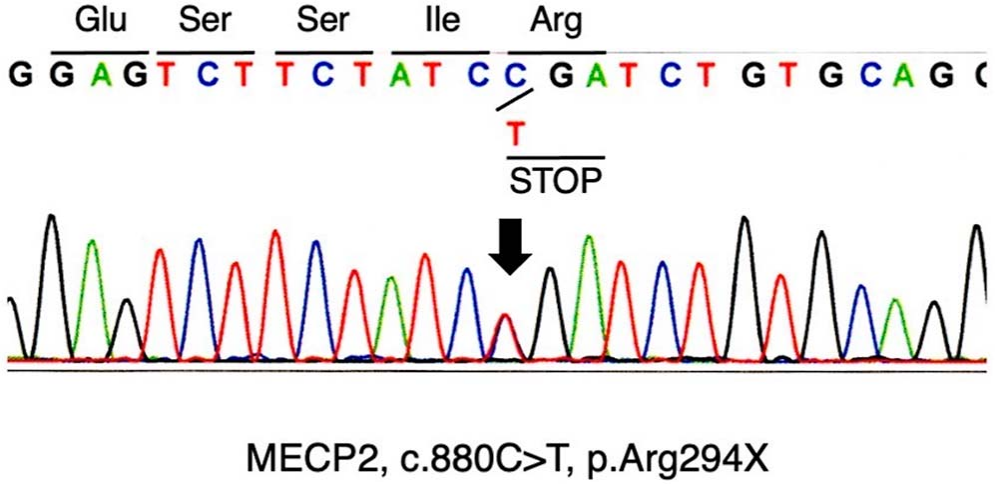
MECP2 sequencing.

The patient was born to non-consanguineous parents. She was delivered at full-term with no noted prenatal or neonatal complications. Her early developmental milestones, such as head control, sitting, crawling and babbling, were not delayed. She spoke her first words at 14 months and walked independently at 18 months. At 3 years of age, she began exhibiting stereotypic hand movements, such as flapping and wringing. At 4 years of age, she was diagnosed with autism spectrum disorder and started on supportive care, including physical and speech therapy. Epilepsy was also diagnosed based on an electroencephalogram and was well controlled with carbamazepine. Brain magnetic resonance imaging revealed no abnormalities. At 10 years of age, genetic testing confirmed the presence of a pathogenic mutation in the methyl-CpG-binding protein 2 gene (MECP2; c.880C > T, p.Arg294*) (Fig. 1). She met several of the 2010 diagnostic criteria for RS (1), including breathing disturbances when awake, bruxism when awake, abnormal muscle tone, inappropriate laughing and intense eye communication, and was diagnosed with RS.

## Discussion

We present the case of a young female with RS who was diagnosed with DM type 1. Table 1 shows the characteristics of previously published cases of the co-occurrence of RS and DM type 1 (2, 3, 4, 5), including our case. Most typical RS patients have pathogenic mutations in MECP2, which is located in Xq28 (1). However, only our case reported the presence of MECP2 genetic mutations. DM type 1 has been reported to be possibly associated with a variety of genetic diseases, such as Down, Turner, Noonan and Klinefelter syndromes (6). Although the association between RS and DM type 1 may be contingent, the accumulation of more cases and investigation of their genetic profiles is worthwhile.

**Table 1:** Characteristics of previously published cases of co-occurrence of RS with DM type 1.

Reference	Country	Age (years)	Gene mutation	Family history of DM	Laboratory data						
					Autoantibody	HbA_1c_ (%)	BG	Blood gas			C-peptide level
								pH	Bicarbonate		
Cameron *et al.* (2)	UK	9	NR	None	NR	NR	46.1 mmol/L	7.67	16 mmol/L	Arterial	Low, serum
Kurtoglu *et al.* (3)	Turkey	4	NR	NR	NR	13.6	237 mg/dL	Normal	NR	Venous	Low, serum
Rekik *et al.* (4)	Tunisia	23	NR	None	GAD, positive	NR	16.8 mmol/L	7.27	5 mmol/L		NR
Akin *et al.* (5)	Turkey	9	NR	None	NR	8.4	529 mg/dL	Normal	NR	Venous	Low, serum
Present study	Japan	3	*MECP2*	None	GAD, positive	10.6	1,091 mg/dL	7.354	14.1 mmol/L	Venous	Low, urine

BG, blood glucose; DM, diabetes mellitus; GAD, anti-glutamic acid decarboxylase antibody; NR, not reported.

When a patient with RS develops diabetic ketoacidosis, it may be characterized by mild acidosis or a normal pH despite the presence of hyperglycemia and dehydration. Among the five patients, only one had acidosis (pH < 7.30) (4). Our patient did not have acidosis despite hyperglycemia and ketonuria. This may be due to compensation by breathing disturbances, which are characteristic of patients with RS. Notably, irregular breathing has been reported in approximately two-thirds of patients with RS (7).

The five cases presented in the literature show an uneven geographic distribution. Ours is the first case from East Asia, whereas the others are from Turkey, the United Kingdom and Tunisia. RS is believed to occur ubiquitously in various racial and ethnic groups worldwide. Although the incidence of DM type 1 by continent is 20 per 100,000 population in the United States, 8 per 100,000 in Africa, 15 per 100,000 in Europe and 15 per 10,000 in Asia (8), we could not confirm any reports of DM type 1 complicated by RS in the United States. Further research is required to elucidate this.

DM has been reported to frequently occur in families with RS (9). However, none of the five cases had a family history of DM and the reason for this remains unclear.

In conclusion, this is the first case report of RS with a confirmed MECP2 mutation complicated by DM type 1. Our case report contributes to the increasing evidence supporting the potential association between RS and DM type 1. Further investigation of genetic mutations in cases of RS complicated by DM type 1 may provide insights into the pathogenesis of these diseases.

## Declaration of interest

The authors declare that there is no conflict of interest that could be perceived as prejudicing the impartiality of the research reported.

## Funding

This work was supported by the Health, Labor and Welfare Sciences Research Grants of Research on rare and intractable diseases (23FC1012). The funder had no role in the design, data collection, data analysis and reporting of this study.

## Patient consent

Written informed consent was obtained from the patient/patient’s mother for publication of this case report.

## Author contribution statement

YK contributed to initial conceptualization, clinical data collection and drafting of the manuscript. ST contributed to genetic analysis and revised the manuscript. All authors read and approved the final version of the manuscript.

## References

[1] Neul JL, Kaufmann WE, Glaze DG, et al. Rett syndrome: revised diagnostic criteria and nomenclature. Ann Neurol 2010 68 944–950. (10.1002/ana.22124) 21154482 PMC3058521

[2] Cameron FJ, Hawkins KC, Khadilkar VV, et al. Insulin-dependent diabetes mellitus presenting with ketoalkalosis in Rett syndrome. Diabet Med 1997 14 884–885. (10.1002/(sici)1096-9136(199710)14:10<884::aid-dia453>3.0.co;2-#) 9371483

[3] Kurtoglu S, Atabek ME, Kumandas S, et al. Diabetes mellitus type 1: association with Rett syndrome. Pediatr Int 2005 47 90–91. (10.1111/j.1442-200x.2005.02018.x) 15693874

[4] Rekik NM, Kamoun M, Mnif F, et al. Type 1 diabetes mellitus and Rett syndrome: is there a link? J Endocrinol Invest 2010 33 851. (10.1007/bf03350352) 21293173

[5] Akin L, Adal E, Akin MA, et al. A case of diabetes mellitus associated with Rett syndrome. J Pediatr Endocrinol Metab 2012 25 197–198. (10.1515/jpem.2011.337) 22570976

[6] Kota SK, Meher LK, Jammula S, et al. Clinical profile of coexisting conditions in type 1 diabetes mellitus patients. Diabetes Metab Syndr 2012 6 70–76. (10.1016/j.dsx.2012.08.006) 23153973

[7] Mackay J, Downs J, Wong K, et al. Autonomic breathing abnormalities in Rett syndrome: caregiver perspectives in an international database study. J Neurodev Disord 2017 9 15. (10.1186/s11689-017-9196-7) 28465761 PMC5410057

[8] Mobasseri M, Shirmohammadi M, Amiri T, et al. Prevalence and incidence of type 1 diabetes in the world: a systematic review and meta-analysis. Health Promot Perspect 2020 10 98–115. (10.34172/hpp.2020.18) 32296622 PMC7146037

[9] Murphy M, Naidu S, Moser HW, et al. Rett syndrome-observational study of 33 families. Am J Med Genet 1986 25 73–76. (10.1002/ajmg.1320250508) 3087205

